# The incidence of malignancy in the residual rectum of IBD patients after colectomy: a systematic review and meta-analysis

**DOI:** 10.1007/s10151-023-02762-w

**Published:** 2023-03-12

**Authors:** I. Georganta, S. McIntosh, D. Boldovjakova, C. N. Parnaby, A. J. M. Watson, G. Ramsay

**Affiliations:** Health Services Research Unit, University of Aberdeen, Foresterhill, Aberdeen, AB252ZD UK

**Keywords:** Inflammatory bowel disease, Rectal stump, Ileal–rectal anastomosis, Rectal malignancy

## Abstract

**Background:**

Patients with inflammatory bowel disease (IBD) who have had a total colectomy remain with their rectum in situ, and are therefore at risk of rectal carcinoma. It is not clear how high the incidence of rectal cancer is in this cohort. The primary objective of this meta-analysis was to estimate the incidence of rectal cancer in patients with ulcerative colitis or Crohn’s disease who have undergone colectomy but have a residual rectum, and to identify the risk factors for its development. In doing so, we explore the current recommendations for screening processes for these patients.

**Methods:**

A systematic review of the literature was performed. Five databases (Medline, Embase, Pubmed, Cochrane Library and Scopus) were searched from inception to 29 October 2021, to identify studies adhering to the population, intervention, control and outcomes (PICO) criteria. The included studies were critically appraised, and the relevant data was extracted. Cancer incidence was estimated from the reported information. Risk stratification was analysed using RevMan. A narrative approach was undertaken for the exploration of the existing screening guidelines.

**Results:**

Data from 23 of the 24 identified studies was suitable for analysis. The pooled incidence of rectal carcinoma was calculated to be 1.3%. Subgroup analysis showed an incidence of 0.7% and 3.2% for patients with a de-functioned rectal stump and ileorectal anastomosis, respectively. Patients with a history of a colorectal carcinoma were more likely to have a subsequent diagnosis of rectal carcinoma (RR 7.2, 95% CI 2.4–21.1). Patients with previous colorectal dysplasia were also at higher risk (RR 5.1, 95% CI 3.1–8.2). No universal standardised guidance regarding screening for this cohort could be identified in the available literature.

**Conclusions:**

The overall risk of malignancy was estimated to be 1.3%, which is lower than previously reported. There is a need for clear and standardised screening guidance for this group of patients.

**Supplementary Information:**

The online version contains supplementary material available at 10.1007/s10151-023-02762-w.

## Introduction

Inflammatory bowel diseases (IBD) are conditions with multifaceted, unclear aetiology, and are associated with dysregulation of the immune system that primarily affects the gastrointestinal tract [1, 2]. Long-term complications for IBD patients include an increased risk of colorectal cancer (CRC) [3, 4]

Despite an increasing number of medical therapies [5], surgery remains a mainstay in the management of IBD [6] and 25–35% of patients with IBD will require surgical management during their lifetime [6]. One common procedure in this context is a total abdominal colectomy. After colectomy, the remaining rectum may be stapled off and left in situ or an ileorectal anastomosis (IRA) can be formed.

The British Society of Gastroenterology (BSG) and the Association of Coloproctology of Great Britain and Ireland (ACPGBI) have published comprehensive guidance on bowel surveillance of patients with IBD to detect CRC early, and therefore optimise outcomes and survival [7, 8]. However, this guidance concentrates on patients with an intact colon and there is little available evidence for screening the rectum of patients who have had a colectomy.

The risk of rectal cancer in such patients remains unclear. Previous assessments have estimated the incidence of malignancy to be around 3% [9]. However, this was before current management strategies for IBD were available and it remains unclear if the incidence rate of CRC cancer in IBD patients has changed over time.

The aim of this systematic review and meta-analysis was to provide a synthesis of the available literature to estimate the incidence rate of CRC in patients with a rectal stump after a colectomy for IBD. We also identified risk stratification for such cases and explored the surveillance strategies for the early identification of malignancy.

## Materials and methods

### Study design

This systematic review and meta-analysis was performed to estimate the incidence of malignancy in the rectal remnant. This study is in line with the Preferred Reporting Items for Systematic Reviews and Meta-Analyses (PRISMA) [10] and Assessing the methodological quality of systematic reviews (AMSTAR) guidelines [11].

### Patient inclusion criteria

The population is patients with a history of IBD who have had a colectomy leaving them with a residual rectal stump or ileorectal anastomosis (IRA). Where appropriate, IBD patients who had a colectomy for CRC or dysplasia were compared with those without either condition. Outcomes were rates of colorectal cancer or descriptors of surveillance regimen published for the early detection of cancer in this setting. Population, intervention, control and outcomes (PICO) criteria are presented in Table 1.

**Table 1:** Summary of population, intervention, control and outcome of the study

	Inclusion	Exclusion
Population	Confirmed diagnosis of Crohn’s disease, ulcerative colitis, or IBD indeterminate colitis Undergone a total colectomy Must have a rectal stump or ileal–rectal anastomosis in situ	Unconfirmed diagnosis Other type of colectomies such as right/left hemicolectomy or Hartman’s -Ileal–anal pouch in situ Patients with a known history of syndromes linked with neoplasia such as familial adenomatous polyposis or Lynch syndrome
Intervention	Past medical history of histologically confirmed colorectal cancer Past medical history of histologically confirmed dysplasia of the colon or the rectum Dysplasia of any stage was included in the review	Past medical history of lesions that have not been confirmed to be malignant History of benign growths at the colon and rectum such as polyps
Control	No past medical history of histologically confirmed dysplasia, or malignancy of the colon or the rectum	No control
Outcome	Malignancy incidence Risk stratification Surveillance regimens	Symptom recurrence Dysplasia incidence

*IBD* inflammatory bowel disease

### Search strategy

A systematic literature search was performed. Five databases: Medline, Embase, Pubmed, Cochrane Library and Scopus were searched from inception to 29 October 2021. Keywords used in the search terms were ‘Crohn’s disease’, ‘Ulcerative Colitis’, ‘Cancer’, ‘Rectal Stump’ and ‘Ileorectal Anastomosis’. The full search strategy for each database is outlined in Supplementary Table 1.

**Fig. 1:**
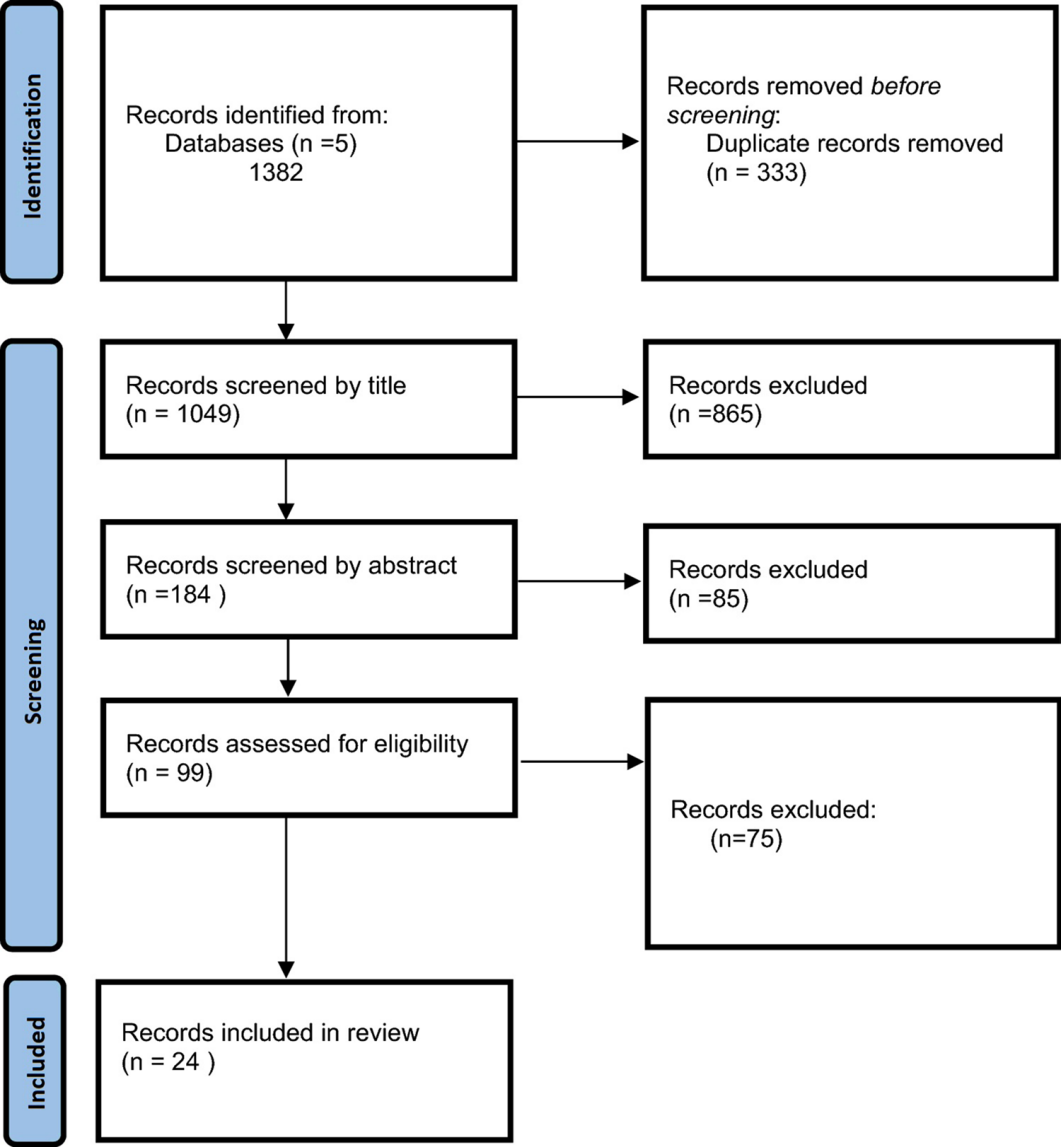
PRISMA flowchart: Preferred Reporting Items for Systematic Reviews and Meta-Analyses

Following completion of the literature search, the studies were exported to the Rayyan software (Rayyan Systems Inc., Qatar) [12]. Duplicate studies were removed and studies were screened in a three-stage process; first by title, then by abstract and finally by full text. Studies were screened by two independent researchers (I.G. and S.M.) and any conflict was resolved by a third reviewer (D.B.). A PRISMA flowchart of the study screening process is displayed in Fig. 1 [10].

### Inclusion criteria

(1) Peer reviewed published manuscripts that reported information on the incidence rates, surveillance techniques or risk factors for malignancy post IBD colectomy. (2) Papers describing the operation as total abdominal colectomy, total colectomy or subtotal colectomy were included due to the variation in definitions. (3) Retrospective, observational and population-based cohort studies and patient series were all included due to the generally low numbers of publications in this field. (4) Only studies published in English language and with at least 20 participants.

### Exclusion criteria

(1) Studies including patients without a confirmed diagnosis of IBD. (2) patients with diagnoses of syndromes related to CRC such as familial adenomatous polyposis (FAP) or Lynch syndrome. (3) Colectomy procedures undertaken for diagnoses other than IBD. (4) Conference abstracts and studies (all small single-centre cohort studies, the largest of which had 42 patients) that were not available in the English language were excluded.

### Primary and secondary outcomes

The primary outcomes of this systematic review were to estimate the published prevalence and incidence rates of malignancy in the residual rectum. The secondary outcome included identification of cohorts of patients at higher risk of rectal malignancy. Additionally, we reviewed any screening regimens from the available literature.

### Data extraction

The data points relevant for analysis were agreed by the members of research group and each individual paper was explored to extract the relevant data. Data were stored and analysed on a Microsoft Excel spreadsheet (Microsoft, USA).

### Critical appraisal

The studies were evaluated according to the Critical Appraisal Skills Programme criteria for cohort studies Checklist (CASP) 2018 [13] by two independent researchers (G.I. and M.S.). The criteria were used to examine for sources of bias, to evaluate the internal validity and to assess the reliability of the evidence. The results were recorded on a table using Word (Microsoft, USA).

### Statistical analysis

Where appropriate, the statistical analysis was conducted in Review Manager (RevMan) V5.4.1 (the Cochrane Collaboration, UK) [14]. Pooled analysis, prevalence and median values were calculated using Excel (Miscrosoft, USA). For the outcome of risk stratification, a dichotomous analysis was undertaken by calculating the risk ratio (RR) with 95% confidence interval (CI). Heterogeneity between the studies was examined by *I*^2^ statistics [15]. A sensitivity analysis was performed for outcomes with significant heterogeneity.

### Study registration

The study was registered in the Research Registry (reviewregistry1370).

## Results

### Study characteristics

In total, 1049 papers were screened and 24 studies were eligible for inclusion (Fig. 1) [16–39, 46]. The earliest paper included was published in 1977 and the most recent in 2021. There were 22 retrospective and two prospective studies. Of the included studies, three were population based and the remaining 21 were patient series. A total of four studies were multicentre, while the remaining 20 were single centre. Fourteen studies were undertaken in Europe (including seven in the UK), three in the USA, two in Asia and one in Australia. The follow-up ranged from 1.9 to 40 years. The study characteristics are described in Table 2.

**Table 2:** Study characteristics

Study	Location	Retrospective (R)/ Prospective (P)	Type	Single centre/multicentre	type of remnant	Number of participants	Male (%)	Median age at surgery	Ulcerative colitis (%)	Crohn’s disease (%)	Indeterminate colitis (%)	Median years of follow-up
Mark-Christensen, 2021	Denmark	Retrospective	Population based	M	RS	4703	2341 (49.7)	38	3677 (78.2)	1026 (21.8)	0 (0)	1.9
Hove, 2018	Netherlands	Retrospective	Population based	M	RS	250	98 (39,2)	39	66 (26.4)	167 (66.8)	17 (6.8)	8
Abdalla, 2017	Sweden	Retrospective	Population based	M	IRA	1112	637 (57.2)	40.6	1112 (100)	0 (0)	0 (0)	8.6
Abdalla, 2017	Sweden	Retrospective	Patient series	M	RS	4358	2630 (60.3)	42.1	4358(100)	0 (0)	0 (0)	5.7
Porter, 2017	UK	Retrospective	Patient series	M	RS	61	37 (60.7)	40.5	37 (60.7)	18 (29.5)	6 (9.8)	6.25
Uzzan, 2017	France	Retrospective	Patient series	S	IRA	343	174 (50.7)	33	284 (82.8)	59 (17.2)	0 (0)	10.6
Ishii, 2016	Japan	Retrospective	Patient series	S	IRA	30	18 (58.1)	36.4	30 (100)	0 (0)	0 (0)	18
Andersson, 2013	Sweden	Retrospective	Patient series	S	IRA	105	71 (67.6)	33.3	105(100)	0 (0)	0 ( 0)	5.4
Munie, 2013	USA	Retrospective	Patient series	S	RS	20	10 (50)	47.1	20 (100)	0 (0)	0 (0)	13.8
Shuno, 2011	Japan	Retrospective	Patient series	S	IRA	29	–	–	29(100)	0 (0)	0 (0)	15.6
Moreira, 2010	USA	Retrospective	Patient series	S	IRA	86	46 (53.5)	28	86 (100)	0 (0)	0 (0)	11
Winther, 2004	Denmark	Prospective	Patient series	S	RS	42	19 (45.2)	–	29 (69.0)	13 (31.0)	0 (0)	2.5–4
Yamamoto, 1999	UK	Retrospective	Patient series	S	RS	69	24 (34.8)	28	0(0)	69(100)	0(0)	–
Pastore, 1997	USA	Retrospective	Patient series	S	IRA	90	48 (53.3)	37.7	48 (53.3)	42 (46.7)	0 (0)	6.5
Khubchandani, 1994	USA	Retrospective	Patient series	S	IRA	129	79 (61.3)	–	60 (46.5)	69 (53.5)	0 (0)	22
Leijonmarck, 1990	Sweden	Retrospective	Patient series	S	IRA	51	23 (45.1)	32	0 (0)	51 (100)	0 (0)	13
Thomas, 1989	UK	Retrospective	Patient series	S	IRA	104	54 (52.0)	–	104 (100)	0 (0)	0 (0)	5
Oakley, 1985	USA	Retrospective	Patient series	S	IRA	145	85 (58.6)	30.4	145 (100)	0 (0)	0 (0)	–
Johnson, 1983	Australia	Retrospective	Patient series	S	IRA	50	–	–	50(100)	0(0)	0(0)	–
Ehsanullah, 1985	UK	Retrospective	Patient series	S	IRA	171	–	–	171 (100)	0 (0)	0 (0)	Up to 40
Grundfest, 1981	USA	Retrospective	Patient series	S	IRA	84	51 (60.7)	29.3	84 (100)	0 (0)	0 (0)	7.7
Farnell, 1980	USA	Retrospective	Patient series	S	IRA	143	69 (48.3)	–	63 (44.1)	80 (55.9)	0 (0)	8.3
Jones, 1978	UK	Retrospective	Patient series	S	IRA	36	19 (52.8)	–	32 (88.9)	4 (11.1)	0 (0)	1–18
Baker, 1978	UK	Retrospective	Patient series	S	IRA	374	172 (46.0)	36.2	374 (100)	0 (0)	0 (0)	10–24
Watts, 1977	UK	Prospective	Patient series	S	IRA	81	36 (44.4)	33	66 (81.5)	15 (18.5)	0	3.5

*IRA* ileorectal anastomosis, *RS* rectal stump

### Participant characteristics

A total of 12,666 patients were included across the 24 studies. The number of participants in each study ranged from 20 to 5470. There were 11,030 patients diagnosed with ulcerative colitis, 1613 with Crohn’s disease and 23 with IBD indeterminate colitis. Table 3 provides a summary of the population, intervention, control and outcome of each study.

**Table 3:** Population, intervention, control and outcome (PICO) summary of studies

Study	Population	Intervention	Control	Outcome
Mark-Christensen, 2021	Patients with IBD who underwent total colectomy (sparing rectum) Identified from Danish National Base Registry	130 patients (2.8%) were diagnosed with colon cancer before total colectomy 34 patients were detected with colorectal dysplasia after total colectomy	No control as malignancy cases < 5 with a history of rectal cancer and not specified for anonymity purposes 4673 patients without a past medical history of dysplasia	Primary: Malignancy incidence Secondary: Risk stratification Surveillance regimen
Hove, 2018	Cohort of IBD patients with a diverted rectum (rectal stump) in a tertiary referral centre using a nationwide registry Patients with continuity of faecal stream (IRA, IPAA) and patients with FAP or Lynch were excluded from the study	-N/A	N/A	Primary: Malignancy incidence Secondary: Surveillance
Abdalla, 2017	-Patients with ulcerative colitis and a colectomy Cohort included patients with diverted rectal stump, IRA and IPAA Data on patients with IPAA was excluded in the review	249 patients had a history of colorectal carcinoma, out of which four developed a rectal carcinoma 70 patients had a history of dysplasia, out of which three developed a carcinoma	5221 patients did not have a history of colorectal carcinoma, out of which 41 developed a rectal carcinoma 5400 patients did not have dysplasia, out of which 42 developed a rectal carcinoma	Primary: Malignancy incidence Secondary: Risk stratification
Porter, 2017	Patients who underwent a colectomy for preoperative diagnosis of UC, CD or indeterminate colitis from a database Patients with incomplete data were excluded	-N/A	-N/A	Primary: Malignancy incidence
Uzzan, 2017	Patients with IRA who were diagnosed with UC or indeterminate colitis before the time of the subtotal colectomy Patients with a diagnosis of Crohn’s were excluded	12 patients had a history of colorectal carcinoma, out of which four developed a rectal carcinoma 17 patients had a history of dysplasia, out of which three developed a rectal carcinoma	331 patients had no history of colorectal carcinoma, out of which 15 developed a rectal carcinoma 326 patients had no history of dysplasia, out of which 16 developed a rectal carcinoma	Primary: Malignancy incidence Secondary: Risk stratification
Ishii, 2016	120 patients with ulcerative colitis underwent either IRA or IPAA and also received endoscopy after the surgery In the systematic review, the patients with an IPAA were not included	N/A	N/A	Primary: Malignancy incidence Secondary: Surveillance
Andersson, 2013	253 consecutive patients with a diagnosis of ulcerative colitis who were operated in a tertiary referral hospital with either IRA or IPAA Patients with IPAA were excluded from the systematic review	Four patients had a history of colorectal carcinoma, out of which one developed a rectal carcinoma	101 patients had no history of colorectal carcinoma, out of which one developed a rectal carcinoma	Primary: Malignancy incidence Secondary: Risk stratification Surveillance
Munie, 2013	Consecutive patients who underwent colectomy with ileostomy for histopathologically proven ulcerative colitis Cases of Crohn’s or indeterminate colitis were excluded based on pathology reports. Patients who underwent completion proctectomy with IPAA at any time after colectomy were also excluded	N/A	N/A	Primary: Malignancy incidence
Shuno, 2011	97 ulcerative colitis patients who received colectomy and postoperative surveillance endoscopy were retrospectively analyzed 29 received IRA, 68 IPAA IPAA patients were excluded from the systematic review	N/A	N/A	Primary: Malignancy incidence Secondary: Surveillance
Moreira, 2010	Patients were identified using a database 86 patients with ulcerative and indeterminate colitis who underwent IRA following total colectomy Patients with Crohn’s disease before the IRA were excluded	N/A	N/A	Primary: Malignancy incidence Secondary: Surveillance
Winther, 2004	54 patients with IBD who had undergone colectomy with terminal ileostomy were recruited from Herlev University Hospital Ten patients refused to participate and two patients were excluded owing to pregnancy and residency abroad	N/A	N/A	Primary: Malignancy incidence
Yamamoto, 1999	The medical records of 69 patients with Crohn’s disease were treated by total colectomy and end ileostomy with a rectal stump	N/A	N/A	Primary: Malignancy incidence
Pastore, 1997	90 patients who underwent total colectomy and ileostomy for UC (48) or CD (42) at Mayo Clinic and Mayo Foundation, Rochester, Minnesota were included	Eight patients had a history of colorectal dysplasia, out of which one developed a rectal carcinoma	24 patients had no history of dysplasia and none of them developed rectal carcinoma	Primary: Malignancy incidence Secondary: Risk stratification
Khubchandani, 1994	Retrospective analysis of 255 patients who underwent colectomy for inflammatory bowel disease 144 patients with IRAs were included in the study	N/A	N/A	Primary: Malignancy incidence Secondary: Surveillance
Leijonmarck, 1990	51 patients with ulcerative colitis who underwent ileorectal anastomosis Resected colon was retrospectively examined Patients with Crohn’s disease were excluded	N/A	N/A	Primary: Malignancy incidence
Thomas, 1989	104 patients for the period 1981–1986	N/A	N/A	Primary: Malignancy incidence
Oakley, 1985	145 patients with ileal–rectal anastomosis for mucosal ulcerative colitis performed at the Cleveland clinic	Six patients had a history of colorectal carcinoma, out of which three developed a rectal carcinoma	135 patients had no history of colorectal carcinoma, out of which two developed a rectal carcinoma	Primary: Malignancy incidence Secondary: Risk stratification
Ehsanullah, 1985	171 patients with a confirmed diagnosis of ulcerative colitis, existing follow-up rectal biopsies with a total colectomy and ileorectal anastomosis	N/A	N/A	Secondary: Surveillance
Johnson, 1983	50 patients with ulcerative colitis were managed by ileorectal anastomosis	33 patients had a history of colorectal dysplasia, out of which five developed a rectal carcinoma	17 patients had no history of colorectal dysplasia, out of which none developed a rectal carcinoma	Primary: Malignancy incidence Secondary: Risk stratification
Grundfest, 1981	89 patients who had an ileal–rectal anastomosis for extensive mucosal ulcerative colitis Patients with granulomatous (transmural) colitis and nonspecific colitis were excluded from the study Pathologic material was reviewed by one pathologist	15 patients had a history of colorectal dysplasia, out of which two developed a rectal carcinoma	69 patients had no history of colorectal dysplasia, out of which two developed a rectal carcinoma	Secondary: Risk stratification
Farnell, 1980	143 patients who underwent abdominal colectomy with primary ileorectal or ileosigmoid anastomosis for chronic ulcerative colitis or Crohn’s disease	N/A	N/A	Primary: Malignancy incidence
Jones, 1978	86 patients who required total colectomy for inflammatory bowel disease, 36 of them had an ileal–rectal anastomosis and were included in the review	N/A	N/A	Primary: Malignancy incidence Secondary: Surveillance
Baker, 1978	The case records of all patients treated by colectomy and ileorectal anastomosis at the Gordon Hospital in the years 1952–1976 374 operation survivors were followed up	Five patients had a history of colorectal carcinoma, out of which one developed a rectal carcinoma	369 patients had no history of colorectal carcinoma, out of which 21 developed a rectal carcinoma	Primary: Malignancy incidence Secondary: Risk stratification
Watts, 1977	81 patients who had an ileorectal anastomosis between 1956 and 1968. The patients were interviewed and examined	N/A	N/A	Primary: Malignancy incidence

*IBD* inflammatory bowel disease, *UC* ulcerative colitis, *IRA* ileorectal anastomosis, *IPAA* ilealpouch–anal anastomosis

### Rate of rectal remnant malignancy

Data on the occurrence of rectal remnant malignancy was available from 23 papers. One study (Ehsanullah [33] had an overlapping population with Baker [37]. Therefore, Ehsanullah was only included in the surveillance outcome.The mean duration of follow-up varied between 2 and 20 years. The mean age at the time of the surgery was 35.6 years. The pooled incidence of rectal malignancy post IBD colectomy was 1.3% from a total of 12,424 patients (median: 1.9%; range: 0.0–10.0%). Table 4 lists the rates of rectal cancer in these patients, by publication.

**Table 4:** Pooled incidence of malignancy in patients with a rectal stump and IRA

Author	Year	Type	Participants	Malignancy	Malignancy Rate (%)
Mark-Christensen	2021	RS	4703	30	0.6
Hove	2018	RS	191	8	4.2
Porter	2017	RS	61	1	1.6
Abdalla	2017	IRA	1112	20	1.8
Abdalla	2017	RS	4358	25	0.6
Uzzan	2017	IRA	343	19	5.5
Ishii	2016	IRA	30	2	6.7
Munie	2013	RS	20	2	10.0
Andersson	2013	IRA	105	2	1.9
Shuno	2011	IRA	29	2	6.9
Moreira	2010	IRA	86	7	8.1
Winther	2004	RS	42	0	0.0
Yamamoto	1999	RS	69	1	1.4
Pastore	1997	IRA	90	1	1.1
Khubchandani	1994	IRA	129	2	1.6
Leijonmarck	1990	IRA	51	1	2.0
Thomas	1989	IRA	104	5	4.8
Oakley	1985	IRA	145	5	3.4
Johnson	1983	IRA	50	5	10.0
Grundfest	1981	IRA	84	4	4.8
Farnell	1980	IRA	143	0	0.0
Jones	1978	IRA	24	0	0.0
Baker	1978	IRA	374	22	5.9
Watts	1977	IRA	81	0	0.0
Total			12,424*	164**	Mean: 1.3
					Range: 0.0–10.0% Median: 1.9

*Total number of participants, **Total number of malignancy cases

The papers were published across a 44-year time frame. The differences in rates of malignancy across the time frame were investigated by calculating the malignancy rate for each paper published in chronological order. In doing this, we noted the lowest rates of malignancies were reported in the studies between 2011 and 2021. Supplementary Table 2 summarises the rates of malignancy by decade.

### Rate of rectal malignancy in the rectal remnant in patients with ulcerative colitis (UC) and Crohn’s disease (CD)

A subgroup analysis was performed in studies that separated the subtypes of IBD into UC and CD (15 studies). Rectal malignancy in patients with UC was available from 13 studies. A total of 6881 patients were included, of whom 108 developed a rectal carcinoma. The pooled rate was 1.6% across the studies (range: 0.0–10.0%; median: 4.8%). Supplementary Table 3 summarises the rates of malignancy. A further subgroup analysis showed a pooled rate of 3.2% in 2503 UC patients with IRA available from 12 studies, and 0.6% in 4360 patients with a rectal stump reported in 2 studies. It refers to subgroup analysis examining Rectal Stump patients with UC which only involves Abdalla and Munie. Unfortunately the rest of the studies with Rectal Stump patients could not be included within this subgroup analysis (eg Hove, Porter) as they included patients from both subgroups (UC, CD), without specifying in which of these subgroups the patients with malignancy belonged to. Upon reviewing, the total number of patients is 4378 from 4360 (4358 Abdalla + 20 Munie).

Studies not reporting if the patients with cancer occurrence belonged in the UC or CD subgroup, were not included in this analysis.

 The remaining two studies examining CD patients included 120 patients, and therefore not deemed sufficient for a pooled analysis

### Prevalence of rectal stump and ileorectal anastomosis malignancy

A subgroup analysis was also performed on the malignancy rates within a de-functioned rectal stump (Supplementary Table 4) and in those with an IRA (Supplementary Table 5). Patients who had a de-functioned rectal stump were identified in seven papers (a total of 9444 patients). The pooled diagnosis rate was 0.7% (median: 1.4%; range: 0.0–10.0%,). A cumulative malignancy incidence of patients with a rectal stump was reported in two studies [16, 18] including 9061 patients. The weighted combined incidence was 0.3% at 10 years post surgery.

IRA patients were assessed in 17 papers with a total of 2980 patients. The pooled prevalence was 3.3% (range: 0.0–10.0%; median 3.4%). A cumulative malignancy incidence of patients with IRA was reported in four studies [18, 20, 21, 25] including 1571 patients. The weighted combined incidence was found to be 2% at 10 years, and 6.8% at 20 years post surgery.

### Pooled incidence of malignancy in the rectal remnant

Pooled incidence was calculated with data from 16 studies [16–25, 29–31, 34, 35, 38] and 11,594 participants. Eleven studies [20–22, 24, 25, 29–31, 34, 35, 38] involved participants with IRA, four studies involved patients with a rectal stump [16, 17, 19, 23]and one included both [18]. The analysis showed that there were 6.5 cases per 100,000 patient-years (Supplementary Table 6).

### Surveillance regimen

Information regarding surveillance was reported in 10 out of the 24 eligible studies. The year of publication of the nine studies ranged from 1985 to 2021. The studies were also geographically varied: three were done in the USA [28, 29, 32], seven in the UK [19, 27, 31, 36–38], two in Japan [21, 23] and three in northern Europe (Sweden [18], the Netherlands [17] and Denmark [16]). Endoscopic investigation was used across all the studies, and seven studies advocated performing biopsies for histological examination [22, 24, 25, 28, 29, 33, 36]. The use of dye spray to identify suspicious lesions in flat mucosa was also reported in one study [24]. In four of the studies, there was no reported data on the frequency of the examination [16, 22, 24, 36]. In two of the publications, the endoscopies were performed annually [21, 25], and in a further two papers, surveillance was between 3 months and 1 year [28, 29].

Finally, one study reported that malignancy was found on magnetic resonance imaging ( MRI), but it is not clear whether the scan was part of the surveillance protocol or if it was performed with a different intention [17]. In the remaining 13 studies, there is no information reported on any surveillance regimen that the population adhered to. Andersson et al. reported that patients underwent surveillance only when they were symptomatic or with a duration of the disease over 10 years [22]. They advise that patient characteristics and risk stratification need to be considered to provide an ideal and personalised screening plan for every individual [22].

No study referenced the use of specific guidelines to optimise the surveillance regimen. One study highlighted significant variability between the type and the interval of screening due to the lack of guidelines, emphasising the importance of standardised guidance [17]. Table 5 provides all the information provided regarding the surveillance regimens followed, by publication.

**Table 5:** Surveillance

Study	Information provided about surveillance regimens
Mark-Christensen, 2021	20 patients involved in surveillance, median time from last surveillance was 1.1 years
Hove, 2018	Although 76% of patients received endoscopic follow-up there was a wide variation in the duration of follow-up and the length of surveillance intervals, most likely due to lack of clear guidelines for this category of patients. There was a total of eight rectal stump cancer cases: four of them were detected with surveillance endoscopy, two with MRI and the remaining two upon removal of the stump
Ishii, 2016	All patients were involved in surveillance, mostly annually
Andersson, 2013	When the symptoms insisted or disease duration was longer than 10 years, the patients underwent endoscopy and biopsies
Shuno, 2011	Meticulous surveillance colonoscopy, using dye spray, biopsies both from suspicious sites and from flat mucosa
Moreira, 2010	Annual proctoscopy and multiple rectal biopsies
Pastore, 1997	Patients with ulcerative colitis were advised to return for a rectal biopsy examination every 6–12 months to check for mucosal dysplasia
Khubchandani, 1994	After surgery, sigmoidoscopy was performed every 3 months and biopsy was performed every 6 months or yearly depending on the findings
Ehsannulah, 1985	79 patients had regular surveillance
Jones, 1978	A sigmoidoscopy and biopsies were performed

*MRI* magnetic resonance imaging

### Risk stratification of malignancy in the rectal remnant

#### History of CRC

**Fig. 2:**
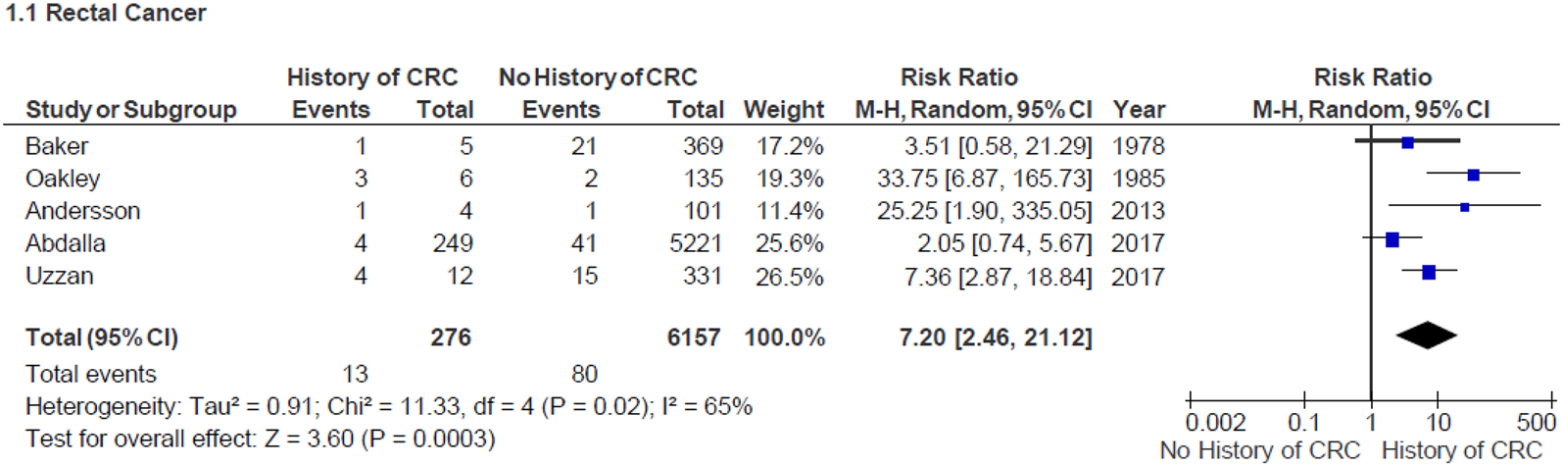
Forest plot: Malignancy occurrence in patients with and without history of colorectal cancer (CRC)

A total of five studies, published between 1978 and 2017, that evaluated a history of colorectal cancer in the colectomy resection as a risk factor for developing malignancy in the residual rectum were included [18, 20, 22, 32, 37]. The number of participants in these studies ranged from 105 to 5470. A total of 6433 patients were examined, of whom 276 had a history of CRC (Fig. 2). Thirteen out of the 276 patients were diagnosed with cancer in the residual rectum (pooled prevalence of 4.7%, Supplementary Table 7). On meta-analysis, CRC patients had a significantly higher risk of synchronous pathology in their rectum than patients without malignancy (RR 7.20, 95% CI 2.46–21.12, *I*^2^ 65%, *p* = 0.0003, Fig. 2).

### History of dysplasia

**Fig. 3:**
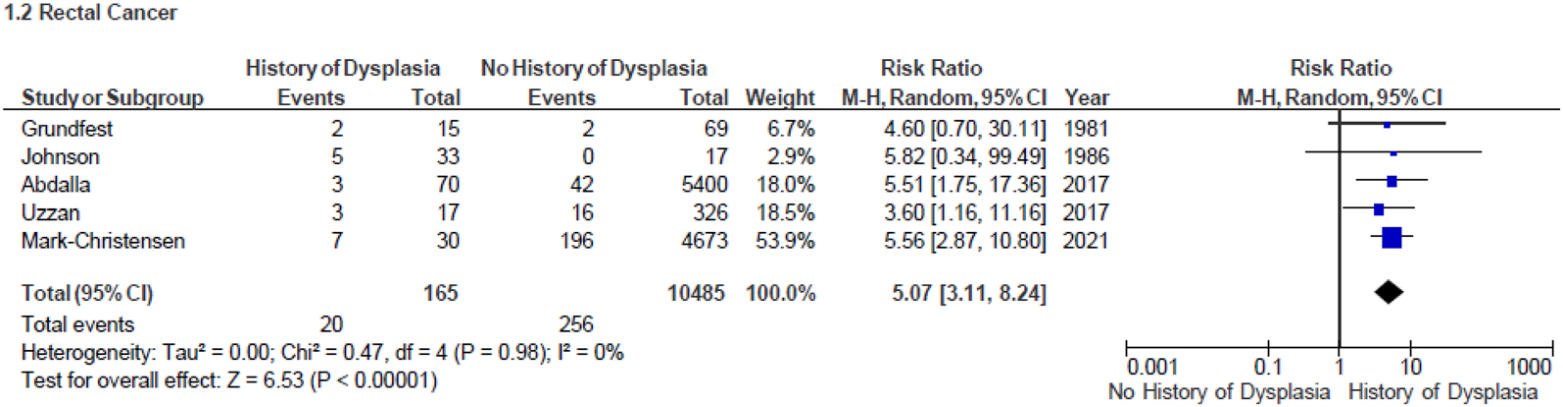
Forest plot: Malignancy occurrence in patients with and without history of dysplasia

Data on a history of dysplasia within the colon was available in five studies published between 1981 and 2021[16, 18, 20, 34, 39]. The number of participants in these studies was between 50 and 5470. They included a total of 10,700 patients, of whom 165 had a history of a biopsy showing dysplasia and 10,485 had no history of dysplasia. Twenty patients out of the 132 with a history of dysplasia were diagnosed with rectal malignancy. Patients with dysplasia were more likely to develop malignancy in a residual rectum compared with patients without a history of dysplasia (RR 5.07, 95% CI 3.11–8.24, *I*^2^ 0%, *p* < 0.0001, Fig. 3).

### Heterogeneity and sensitivity analysis

Heterogeneity was found to be significant (*I*^2^ 65%) on meta-analysis exploring history of colorectal cancer. A sensitivity analysis was performed by removing studies one by one and assessing the effect. One study [18] contributed the majority of the heterogeneity, possibly a result of being a larger cohort than the rest of the included publications. Excluding this study resulted in an increased risk ratio. However, this study was one of the larger studies as it was a multicentre cohort and patients were recruited from a registered database. The study could therefore not be justifiably excluded from the analysis.

### Critical appraisal

The critical appraisal showed that the majority of studies were of low quality. There were no large datasets and most were case series. However, 15 of the 24 studies met at least 10 out of the 11 criteria [16–18, 20–23, 25, 27, 28, 30, 33–35, 37]. Eight studies were positive for 7–9 out of 11 criteria [19, 24, 26, 29, 31, 36, 39, 46], while one of the studies met only six of the criteria [32]. The summary of quality assessment for the 24 included studies is detailed in Supplementary Table 8.

## Discussion

In this systematic review and meta-analysis of the incidence, risk factor stratification and surveillance strategy for rectal malignancy in post total colectomy IBD patients, we have identified some key findings. Firstly, the pooled prevalence of residual rectum malignancy after colectomy across the literature is 1.3%. Interestingly, this is lower than that quoted previously, which has been 3% [9]. Such a finding is key for patient counselling in terms of assessment of the rectum after colectomy, surveillance stratification and decisions regarding further management.

Given that medical management aims to reduce inflammation and that a pro-inflammatory state potentiates malignancy [40], it may be that the lower rates reflect the long-term effects of immuno-biologic medications introduced in the early 2000s. However, it is likely to be many years before we will be able to confirm this hypothesis. It is, nevertheless, intriguing that, in this assessment of rates of malignancy across the 44 years of the publications available, the decade with the lowest rate of cancer detection was the most recent.

A further key finding of this study is that there is standardised screening guidance for this group. We identified a common trend in the reported frequency of surveillance: endoscopic examination performed annually or biannually. The absence of any guidance means that surveillance is currently at the discretion of the clinician, making service provision challenging.

Adhering to screening guidelines that are designed for patients with an intact bowel can result in exposing patients to unnecessary tests that could potentially cause harm and discomfort [41], and such approaches may not be cost effective. There is a further question of accuracy of surveillance. Luminal investigations may be challenging if the rectal stump has been strictured down, preventing adequate visualisation of the upper aspect of the rectal remnant. MRI of the pelvis may be helpful in this setting [42]. However, there are insufficient data from the papers identified in this review to comment further on surveillance.

The pooled prevalence of malignancy in the rectal stump and IRA in this review was 0.7% and 3.2% respectively. Previous literature has reported rates of 2.1% for patients with a de-functioned rectum and 2.4% for IRA patients [9]. Overall, our findings indicate the malignancy risk in these cohorts is still lower than the general population lifetime risk of developing CRC, which is estimated at 4.4% [43]. Given the inherent differences in the rectal stump and IRA patient cohorts it is not possible to comment further on whether this difference in malignancy detection is anything other than differences in the patient cohorts.

A history of CRC was found to be a risk factor, which agrees with existing literature for both the general population with IBD and for IBD patients with total colectomy [9, 44]. An interesting finding of this study is that the pooled prevalence of cancer recurrence after a colorectal primary was 4.7%. In contrast, a recent study published in 2016 reported that 17% of the participants who were treated for CRC with a curative-intent experienced recurrence [45].

It is important to acknowledge that surgical and endoscopic techniques have changed over time, with ileal pouch–anal anastomosis a common surgical procedure which necessitates rectal resection. Consequently, rectal cancer risk is reduced. However, such a procedure is not without risk. Adverse events such as effects on female fecundity and pelvic nerve damage must be taken into account when counselling patients for such procedures [46, 47].

One of the limitations of this study is the large number of low-quality studies and the inclusion of only few large patient cohorts. Larger data could be retrieved from a national registry of IBD management. However, to date, no such registry exists. [48]. Another limitation is that only English language studies were included. However, non-English studies identified on abstract review that could potentially have been eligible were small cohort studies which were unlikely to influence the results.

## Conclusions

The pooled analysis of rectal cancer was reported at 1.3% for IBD patients with both an IRA and a rectal stump. History of colorectal cancer and dysplasia was associated with developing malignancy in the residual rectum. However, this is an understudied area with few large-scale good-quality studies. Furthermore, no consistent guidance for surveillance of this group currently exists.

## Supplementary Information

Below is the link to the electronic supplementary material.

Supplementary file1 (DOCX 36 KB)

## Data Availability

The data that support the findings of this study are openly available in the reference list provided below.

## Declarations

### Research registration

Research registry, unique identifying number: reviewregistry1370.
