# Supplementary material for: The incidence of malignancy in the residual rectum of IBD patients after colectomy: a systematic review and meta-analysis
Source: Tech Coloproctol. 2023 Mar 12;27(9):699–712. doi: 10.1007/s10151-023-02762-w (PMC10404177; doi:10.1007/s10151-023-02762-w)
Supplement: Supplementary file 1 — Supplementary file1 (DOCX 36 KB) [file 10151_2023_2762_MOESM1_ESM.docx]

**SUPPLEMENTARY MATERIAL**

**Supplementary table 1- Search Terms**

Search on 29/10/21

**OVID –** Embase <1996 to 2021 Week 42>, Ovid MEDLINE(R) <1946 to October Week 4 2021>

| Search | Keywords | # of Results |
| --- | --- | --- |
| #1 | exp crohn disease/ | 127,392 |
| #2 | exp ulcerative colitis/ | 105,870 |
| #3 | (crohn* or colitis ulcerative or ulcerative colitis or colitis or indeterminate or indeterminate colitis or IBD or inflammatory bowel disease or UC).af. | 475,146 |
| #4 | 1 or 2 or 3 | 475,146 |
| #5 | (dysplasia or malign* or premalign* or precancer* or pre-cancer* or histologic* or cancer* or neoplasm* or tumour* or tumor* or patholog*).af. | 13,773,817 |
| #6 | exp neoplasms/ | 7,577,876 |
| #7 | 5 or 6 | 14,274,587 |
| #8 | ((rectal and (stump* or remnant*)) or ileorectal anastomosis or IRA or ileal-rectal anastomosis).af. | 29,296 |
| #9 | 4 and 7 and 8 | 871 |
| #10 | remove duplicates from 9 | 721 |
| #11 | limit 10 to human | 682 |

| **Pubmed Search Terms – Results: 277** |
| --- |
| ((IBD OR INFLAMMATORY BOWEL DISEASE OR CROHN* OR "COLITIS ULCERATIVE" OR "ULCERATIVE COLITIS" OR "COLITIS INDETERMINATE" OR "INDETERMINATE COLITIS") AND ( DYSPLASIA OR MALIGN* OR PREMALIGN* OR "PREMALIGN*" OR PRECANCER* OR "PRE-CANCER*" OR HISTOLOGIC* OR CANCER* OR NEOPLASM* OR TUMOUR* OR TUMOR* OR PATHOLOG* ) AND ( "RECTAL STUMP*" OR RECTAL REMANT* OR IRA OR ILEORECTAL ANASTOMOSIS OR ILEAL-RECTAL ANASTOMOSIS)) |

| **Scopus Search Terms – Results: 289** |
| --- |
| TITLE-ABS-KEY ( ( ( *ibd*  OR  *inflammatory*  AND  *bowel*  OR  *crohn**  OR  *"colitis ulcerative"*  OR  *"ulcerative colitis"*  OR  *"colitis indeterminate"*  OR  *"indeterminate colitis"* )  AND  ( *dysplasia*  OR  *malign**  OR  *premalign**  OR  *"pre-malign*"*  OR  *precancer**  OR  *"pre-cancer*"*  OR  *histologic**  OR  *cancer**  OR  *neoplasm**  OR  *tumour**  OR  *tumor**  OR  *patholog** )  AND  ( *"rectal stump*"*  OR  *rectal*  AND  *remnant**  OR  *ira*  OR  *ileorectal*  OR  *ileal-rectal*  OR  *anastomosis*  OR  *residual* ) ) ) |

| **Cochrane Search terms - Results: 134** |
| --- |
| ((IBD OR inflammatory bowel disease OR crohn* OR "colitis ulcerative" OR "ulcerative colitis" OR "colitis indeterminate" OR "indeterminate colitis") AND ( dysplasia OR malign* OR premalign* OR "pre-malign*" OR precancer* OR "pre-cancer*" OR histologic* OR cancer* OR neoplasm* OR tumour* OR tumor* OR patholog* ) AND ( "rectal stump*" OR rectal remnant* OR remnant OR residual colon OR residual OR IRA OR ileorectal OR ileal-rectal OR anastomosis)) in Title Abstract Keyword - (Word variations have been searched) |

***Supplementary table 2:* Data by decade**

| Author | Year | Type | Participants | Malignancy |
| --- | --- | --- | --- | --- |
| Mark-Christensen | 2021 | RS | 4,703 | 30 |
| Hove | 2018 | RS | 191 | 8 |
| Porter | 2017 | RS | 61 | 1 |
| Abdalla | 2017 | IRA | 1,112 | 20 |
| Abdalla | 2017 | RS | 4,358 | 25 |
| Uzzan | 2017 | IRA | 343 | 19 |
| Ishii | 2016 | IRA | 30 | 2 |
| Munie | 2013 | RS | 20 | 2 |
| Andersson | 2013 | IRA | 105 | 2 |
| Shuno | 2011 | IRA | 29 | 2 |
| **Total** | | | **10,952** | **111** |
| **Percentage** | | | **1.01%** | |

| Author | Year | Type | Participants | Malignancy |
| --- | --- | --- | --- | --- |
| Moreira | 2010 | IRA | 86 | 7 |
| Winther | 2004 | RS | 42 | 0 |
| **Total** | | | **128** | **7** |
| **Percentage** | | | **5.47%** | |

| Author | Year | Type | Participants | Malignancy |
| --- | --- | --- | --- | --- |
| Yamamoto | 1999 | RS | 69 | 1 |
| Pastore | 1997 | IRA | 90 | 1 |
| Khubchandani | 1994 | IRA | 129 | 2 |
| **Total** | | | **288** | **4** |
| **Percentage** | | | **1.38%** | |

| Author | Year | Type | Participants | Malignancy |
| --- | --- | --- | --- | --- |
| Leijonmarck | 1990 | IRA | 51 | 1 |
| Lofberg | 1990 | IRA | 15 | 0 |
| Thomas | 1989 | IRA | 104 | 5 |
| Oakley | 1985 | IRA | 145 | 5 |
| Johnson | 1983 | IRA | 50 | 5 |
| Grundfest | 1981 | IRA | 84 | 4 |
| **Total** | | | **449** | **20** |
| **Percentage** | | | **4.45%** | |

| Author | Year | Type | Participants | Malignancy |
| --- | --- | --- | --- | --- |
| Farnell | 1980 | IRA | 143 | 0 |
| Jones | 1978 | IRA | 24 | 0 |
| Baker | 1978 | IRA | 374 | 22 |
| **Total** | | | **541** | **22** |
| **Percentage** | | | **4.07%** | |

***Supplementary table 3 – Pooled incidence of malignancy in the subgroup of patients with UC***

| Study | Type | Participants | Malignancy | Rate (%) |
| --- | --- | --- | --- | --- |
| Abdalla, 2017 | IRA, RS | 5470 | 45 | 0.8 |
| Ishii, 2016 | IRA | 30 | 2 | 6.7 |
| Munie, 2013 | RS | 20 | 2 | 10.0 |
| Andersson, 2013 | IRA | 105 | 2 | 1.9 |
| Shuno, 2011 | IRA | 29 | 2 | 6.9 |
| Moreira, 2010 | IRA | 86 | 7 | 8.1 |
| Lofberg, 1990 | IRA | 15 | 0 | 0.0 |
| Thomas, 1989 | IRA | 104 | 5 | 4.8 |
| Oakley, 1985 | IRA | 145 | 5 | 3.4 |
| Johnson, 1983 | IRA | 50 | 5 | 10.0 |
| Grundfest, 1981 | IRA | 84 | 4 | 4.8 |
| Baker, 1978 | IRA | 374 | 22 | 5.9 |
| Aylett, 1971 | IRA | 369 | 7 | 1.9 |
| **Total** |  | **6881** | **108** | **1.6** |

*Supplementary table 4: Pooled Incidence of malignancy in patients with a rectal stump*

| Study | Participants | Malignancy | Rate (%) |
| --- | --- | --- | --- |
| Mark-Christensen,2021 | 4,703 | 30 | 0.6 |
| Hove, 2018 | 191 | 8 | 4.2 |
| Porter, 2017 | 61 | 1 | 1.6 |
| Abdalla, 2017 | 4,358 | 25 | 0.6 |
| Munie , 2013 | 20 | 2 | 10.0 |
| Winther, 2004 | 42 | 0 | 0.0 |
| Yamamoto,1999 | 69 | 1 | 1.4 |
| **Total** | **9,444** | **67** | **Mean: 0.7** |
|  |  | | **Range:0.0-10.0%**  **Median: 1.4** |

| Author | Year | Type | Participants | Malignancy | Rate(%) |
| --- | --- | --- | --- | --- | --- |
| Abdalla | 2017 | IRA | 1112 | 20 | 1.8 |
| Uzzan | 2017 | IRA | 343 | 19 | 5.5 |
| Ishii | 2016 | IRA | 30 | 2 | 6.7 |
| Andersson | 2013 | IRA | 105 | 2 | 1.9 |
| Shuno | 2011 | IRA | 29 | 2 | 6.9 |
| Moreira | 2010 | IRA | 86 | 7 | 8.1 |
| Pastore | 1997 | IRA | 90 | 1 | 1.1 |
| Khubchandani | 1994 | IRA | 129 | 2 | 1.6 |
| Leijonmarck | 1990 | IRA | 51 | 1 | 2.0 |
| Thomas | 1989 | IRA | 104 | 5 | 4.8 |
| Oakley | 1985 | IRA | 145 | 5 | 3.4 |
| Johnson | 1983 | IRA | 50 | 5 | 10.0 |
| Grundfest | 1981 | IRA | 84 | 4 | 4.8 |
| Farnell | 1980 | IRA | 143 | 0 | 0.0 |
| Jones | 1978 | IRA | 24 | 0 | 0.0 |
| Baker | 1978 | IRA | 374 | 22 | 5.9 |
| Watts | 1977 | IRA | 81 | 0 | 0.0 |
| **Total** | | | **2980** | **97** | **Mean: 3.3** |
|  | | |  | | **Range: 0.0-10.0%**  **Median: 3.4%** |

**Supplementary table 5: Pooled Incidence of malignancy in patients with IRA**

**Supplementary table 6: Malignancy Incidence Rate**

| Author | Year | Type | Participants | Malignancy | Follow up (years) |
| --- | --- | --- | --- | --- | --- |
| Mark-Christensen | 2021 | RS | 4,703 | 30 | 1.9 |
| Hove | 2018 | RS | 191 | 8 | 8 |
| Porter | 2017 | RS | 61 | 1 | 6.25 |
| Abdalla | 2017 | IRA  RS | 1,112 4,358 | 20  25 | 8.6  5.7 |
| Uzzan | 2017 | IRA | 343 | 19 | 10.6 |
| Ishii | 2016 | IRA | 30 | 2 | 18 |
| Munie | 2013 | RS | 20 | 2 | 13.8 |
| Andersson | 2013 | IRA | 105 | 2 | 5.4 |
| Shuno | 2011 | IRA | 29 | 2 | 15.6 |
| Moreira | 2010 | IRA | 86 | 7 | 11 |
| Khubchandani | 1994 | IRA | 129 | 2 | 22 |
| Lofberg | 1990 | IRA | 15 | 0 | 4 |
| Thomas | 1989 | IRA | 104 | 5 | 20.1 |
| Grundfest | 1981 | IRA | 84 | 4 | 7.7 |
| Farnell | 1980 | IRA | 143 | 0 | 8.3 |
| Watts | 1977 | IRA | 81 | 0 | 3.5 |
| **Total** | | | **11,594** | **129** | **170** |
| **Percentage** | | | **0.0111** = **1.1%** | |  |
| **Incidence ( per 100,000 patient years)** | | |  | | **6.53/100,000** |

*Supplementary table 7 – Pooled Incidence of malignancy in patients with a history of CRC*

| Author | Year | Type | Participants with previous CRC | Malignancy |
| --- | --- | --- | --- | --- |
| Abdalla | 2017 | IRA+RS | 249 | 4 |
| Uzzan | 2017 | IRA | 12 | 4 |
| Andersson | 2013 | IRA | 4 | 1 |
| Oakley | 1985 | IRA | 6 | 1 |
| Baker | 1970 | IRA | 5 | 3 |
| **Total** | | | **276** | **13** |
| **Percentage** | | | **4.7%** | |

***Supplementary table 8:*** Critical Appraisal

| Study | Q1 | Q2 | Q3 | Q4 | Q5(a) | Q5(b) | Q6(a) | Q6(b) | Q9 | Q10 | Q11 |
| --- | --- | --- | --- | --- | --- | --- | --- | --- | --- | --- | --- |
| Mark Chistensen | Y | Y | Y | Y | Y | Y | Y | N | Y | Y | Y |
| Hove | Y | Y | Y | Y | Y | Y | Y | Y | Y | Y | Y |
| Porter | Y | Y | N | Y | Y | N | Y | Y | Y | Y | Y |
| Abdalla | Y | Y | Y | Y | Y | Y | Y | Y | Y | Y | Y |
| Uzzan | Y | Y | Y | Y | Y | Y | Y | Y | Y | Y | Y |
| Ishii | Y | Y | Y | Y | Y | Y | Y | Y | Y | C/T | Y |
| Munie | Y | Y | Y | Y | Y | Y | Y | Y | Y | C/T | Y |
| Andersson | Y | Y | Y | Y | Y | Y | Y | Y | Y | Y | Y |
| Shuno | Y | Y | N | Y | N | N | Y | Y | Y | C/T | Y |
| Moreira | Y | Y | Y | Y | Y | Y | Y | Y | Y | C/T | Y |
| Winther | Y | Y | N | Y | N | N | Y | N | Y | Y | Y |
| Yamamoto | Y | Y | Y | Y | Y | Y | Y | Y | Y | Y | Y |
| Pastore | Y | Y | Y | Y | Y | Y | Y | Y | Y | C/T | Y |
| Khubchandani | Y | Y | Y | Y | N | N | Y | Y | Y | C/T | Y |
| Leijonmarck | Y | Y | Y | Y | Y | Y | Y | Y | Y | Y | Y |
| Thomas | Y | Y | N | Y | N | N | Y | Y | Y | Y | Y |
| Johnson | Y | Y | N | Y | N | N | Y | Y | Y | C/T | Y |
| Oakley | Y | Y | N | Y | N | N | C/T | C/T | Y | C/T | Y |
| Ehsannulah | Y | Y | Y | Y | Y | Y | Y | Y | Y | Y | Y |
| Grundfest | Y | Y | Y | Y | Y | Y | Y | Y | Y | C/T | Y |
| Farnell | Y | Y | Y | Y | Y | Y | Y | Y | Y | C/T | Y |
| Jones | Y | Y | N | Y | N | N | Y | Y | Y | Y | Y |
| Watts | Y | Y | Y | Y | Y | Y | Y | Y | Y | C/T | Y |
| Baker | Y | Y | Y | Y | Y | Y | Y | Y | Y | Y | Y |

Key:

Q1 Did the study address a clearly focused issue?

Q2 Was the cohort recruited in an acceptable way?

Q3 Was the exposure accurately measured to minimise bias?

Q4 Was the outcome accurately measured to minimise bias?

Q5 (a) Have the authors identified all important confounding factors?

Q5 (b) Have they taken account of the confounding factors in the design and/or analysis?

Q6 (a)Was the follow up of the subjects complete enough?

Q6 (b) was the follow up of the subjects long enough?

Q9 Do you believe the results?

Q10 Can the results be applied to the local population?

Q11 Do the results of this study fit with other available evidence?

Y = Yes, N = No, C/T = Cannot Tell
